# Fulminant listerial infection of the central nervous system in an otherwise healthy patient: a case report

**DOI:** 10.4076/1752-1947-3-7383

**Published:** 2009-06-30

**Authors:** Dimitrios Karakitsos, George Samonis, Vasilios Georgountzos, Andreas Karabinis

**Affiliations:** 1 Intensive Care Unit, General Hospital of Athens, Mesogeion Avenue, Athens, 11527, Greece; 2 Infectious Diseases Unit, Department of Internal Medicine, University of Crete, Iraklion, Crete, Greece; 3 Radiology Department, General Hospital of Athens, Mesogeion Avenue, Athens, 11527, Greece

## Abstract

**Introduction:**

The mortality of listerial rhombo-encephalitis exceeds 26% and may involve otherwise healthy patients. A case is presented of a man with fatal listerial infection of the central nervous system that was monitored in an intensive care unit.

**Case presentation:**

A 42-year-old, previously healthy man was admitted with fever of 39°C, blurred vision, confusion and headache. He had right-sided central facial paresis, bilateral absent gag reflex and bilateral cerebellar ataxia. After a few hours, he became septic and developed bilateral vocal cord paralysis and airway obstruction. He was intubated and put on mechanical ventilation. Computed tomography brain scans revealed multiple frontal hypodense areas and slight hydrocephalus. Cerebrospinal fluid findings included pleocytosis of 4200 cells/μL (77% neutrophils), protein of 114 mg/dL and normal glucose levels. Listerial infection was suspected; therefore ampicillin was added to his initial therapeutic regimen, already including ceftriaxone and gentamicin. All cultures were negative, and no immunologic abnormality could be documented, but the patient's clinical condition deteriorated rapidly. Continuous neuromonitoring by means of transcranial Doppler and optic nerve sonography along with follow-up computed tomography brain scans confirmed the severity of the brain damage; hence, dexamethasone and mannitol were also administered. The patient was clinically documented as 'brain dead' 7 days after his admission to the intensive care unit; thereafter, blood- and post-mortem brain tissue cultures grew *Listeria monocytogenes*.

**Conclusion:**

This case report illustrates the importance of neuromonitoring in patients with severe brain damage. We also show that, despite prompt antibiotic treatment and dexamethasone administration, listerial infection of the central nervous system can be lethal.

## Introduction

*Listeria monocytogenes* is an anaerobic, Gram-positive bacillus causing infections of the central nervous system (CNS) primarily observed in immunocompromised hosts. A specific form of listerial CNS infection, listerial rhombo-encephalitis, usually occurs in otherwise healthy adults. Typically, it is a biphasic illness with neurologic signs appearing 4 to 5 days after the onset of fever. Mortality exceeds 26%, and serious sequelae are common in survivors [1]. We present a case of listerial CNS infection in a previously healthy person in whom diagnosis was based on the clinical findings of blood- and post-mortem tissue cultures, cerebrospinal fluid (CSF) results and computed tomography (CT). The patient was admitted to the intensive care unit (ICU) and underwent continuous neuromonitoring. Despite early administration of antibiotic and dexamethasone treatment, the listerial infection proved lethal. Previous studies did not focus on the neuromonitoring in cases of CNS infection. In our report, we performed non-invasive methods such as transcranial Doppler sonography (TCD) and optic nerve sonography, which may be used in the ICU for neuromonitoring purposes.

## Case presentation

A 42-year-old right-handed and previously healthy man presented to the emergency department with a fever of 39°C, blurred vision, confusion and headache. On examination, he had decreased right-sided corneal reflex, a right-sided central facial paresis, bilateral absent gag reflex and marked bilateral cerebellar ataxia. After a lumbar puncture was performed, treatment with ceftriaxone was initiated. However, the patient became septic and was subsequently intubated, put on mechanical ventilation and transferred to the ICU due to bilateral vocal cord paralysis and airway obstruction. Gentamicin was also added to his therapeutic regimen due to the rapidly developed sepsis.

**Figure 1:**
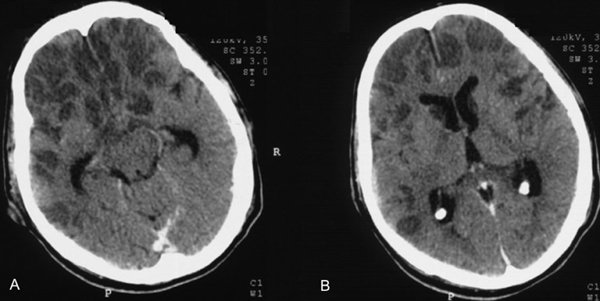
**Brain computed tomography scans depicting frontal multiple hypodense areas **(A)** and a slight hydrocephalus **(B)****.

Upon admission, brain CT scans revealed multiple hypodense areas frontally and slight hydrocephalus (Figure 1). CSF findings included pleocytosis of 4200 cells/μL (77% neutrophils, 22% lymphocytes and 1% monocytes), protein of 114 mg/dL and normal glucose. Based upon the above clinical, laboratory and imaging findings, a listerial infection was suspected; hence ampicillin was added to the initial therapeutic regimen. At that time, CSF cultures were negative for *Listeria* as well as other bacterial, viral and fungal microorganisms. The spinal fluid Veneral Disease Research Laboratory test and the results of tests for Lyme disease antibody, toxoplasmosis titers and cryptococcal antigen were all negative. Results of a serum human immunodeficiency virus test were also negative, and further laboratory investigations failed to confirm any immunological abnormalities.

**Figure 2:**
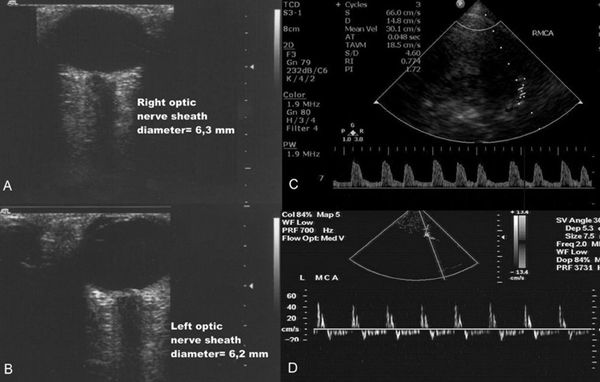
**Optic nerve sonography showed increased optic nerve sheath diameter **(A, B)**; Transcranial Doppler sonography documented increased (1,72) pulsatility index in the right middle cerebral artery **(C)**; the patient was declared 'brain dead' by clinical tests, while transcranial Doppler showed a reversal of diastolic blood flow in the left middlecerebral artery **(D)****.

The patient's clinical condition in the ICU deteriorated in the first 48 hours following admission, despite the aggressive administration of fluids and vasopressors. He developed cardiac tachyarrhythmia and was hemodynamically unstable. Continuous neuromonitoring was performed by means of TCD and optic nerve sonography, as previously described [2]-[4]. We utilized an HDI 3500 ultrasound device (ATL, Philips, Bothell, USA) and a Philips XD11 XE ultrasound device (Philips, Bothell, US), both equipped with a 7.5 MHz linear transducer as well as with 1.5 MHz to 3.6 MHz wide-angle, phased-array transducers. TCD revealed progressively decreased diastolic flow velocities and increased pulsatility index of cerebral blood flow in the middle cerebral artery bilaterally, while the optic nerve sheath diameter was repeatedly found to be increased (Figure 2).

**Figure 3:**
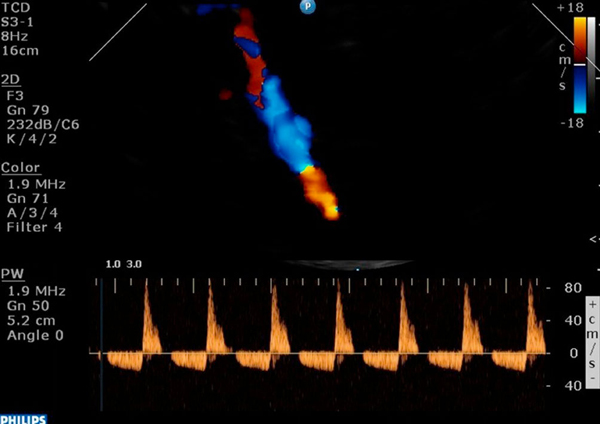
**The patient was declared 'brain dead' by clinical tests, while transcranial Doppler depicted reversal of diastolic blood flow in the left middle cerebral artery; on color mode, systolic and diastolic flow coexisted in the same time unit resulting in a pulsating flash akin to the beacon of a lighthouse**.

The above data gave the impression of severe brain damage and consequently of cerebral edema. Indeed, increased pulsatility index and increased optic nerve sheath diameter correspond to the diagnosis of brain damage [2]-[4]. Furthermore, the above findings were confirmed by a brain CT scan that was performed 3 days after admission and revealed diffuse brain damage and cerebral edema. Hence, dexamethasone and mannitol were added in the therapeutic regimen. At that time, another CSF analysis revealed pleocytosis of 6800 cells/μL (85% neutrophils, 13% lymphocytes and 2% monocytes), protein of 322 mg/dL and glucose of 2 mg/dL. Unfortunately, brain death was diagnosed by pertinent clinical tests 7 days after the patient's admission to the ICU. Interestingly, the TCD revealed a reversal of the diastolic cerebral blood flow (Figure 2). While on color mode, systolic and diastolic flow coexisted in the same time unit, resulting in a pulsating flash akin to the beacon of a lighthouse, as previously described (Figure 3) [see also [4]. Three days after the diagnosis of brain death was established, the patient died. Blood- and post-mortem brain tissue cultures grew *L. monocytogenes*, while the histopathology result showed numerous intracellular and extracellular bacilli in the areas of the brain stem and cerebellar white matter.

## Discussion

Rhombo-encephalitis is a particular form of listerial encephalitis that affects mainly the brain stem and the cerebellum (rhombencephalon) and usually occurs in otherwise healthy adults. A listerial infection may be misdiagnosed because the prodromal symptoms are nonspecific and meningeal signs are uncommon. Brain-stem encephalitis should be considered upon progression of such symptoms as nystagmus, gaze palsy, facial numbness, vertigo, dysphagia, persistent hiccupping and respiratory failure, suggesting multiple cranial nerve involvement. Early diagnosis of brain-stem involvement is essential, as respiratory failure can lead to death if untreated. The CSF in a listerial infection typically reveals an increased leukocyte count, usually with a predominance of polymorphonuclear cells, increased protein and normal glucose levels [5]. *L. monocytogenes* is difficult to isolate from the CSF but is often readily cultured from blood. In this patient, the microorganism grew in blood- and post-mortem brain tissue cultures, while the CSF cultures were negative.

There are only a few studies of listerial infection with imaging data and none with neuromonitoring information. Most of the reports of listerial rhombo-encephalitis have described normal findings on CT scans [6]-[9]. Some abnormal CT-scan findings in documented listerial infections have included widening of the brain stem, hydrocephalus, brain-stem or cerebellar abscess, and vermian hemorrhage [9,10]. In our patient, continuous neuromonitoring in the ICU setting by means of TCD and optic nerve sonography gave the clinicians the impression that his cerebral microcirculation was compromised due to the severity of brain damage, and that a diffuse brain edema was present, corresponding to an increased optic nerve sheath diameter [2]-[4]. The above findings were confirmed by a pertinent brain CT scan. The patient received aggressive antibiotic treatment with ceftriaxone, ampicillin and gentamicin. Furthermore, dexamethasone was added to his therapeutic regimen because of its potent anti-inflammatory activity and its role in controlling cerebral edema [11,12]. Anecdotal reports have indicated that dexamethasone might be useful in listerial CNS infections; however, this was not confirmed in our patient [13]. Unfortunately, the patient was declared 'brain dead' 7 days after his admission in the ICU by usual clinical tests; furthermore, cessation of cerebral circulation was confirmed by TCD findings [4].

There are only some data available concerning the neuromonitoring of patients who present with severe CNS infections. However, in our patient, prompt neuromonitoring by means of TCD and optic nerve sonography provided important information concerning the severity of the CNS damage and consequent cerebral edema. Hence, the above non-invasive measures may be of clinical value in the neuromonitoring of severe CNS infections in the ICU setting. Finally, in the presence of an acute onset of progressive cranial nerve dysfunction and ataxia, and of CSF leukocytosis with polymorphonuclear cell predominance with normal glucose levels, listerial CNS infection should always be suspected. Early treatment can decrease the morbidity and mortality of this rare pathogen. Our results indicate that administration of dexamethasone is of questionable benefit, especially if focal neurologic signs are present. Also, the present results were in accordance with past studies which suggest that fatal cases of listerial rhombo-encephalitis may be observed in healthy patients and may be associated with neuropathologic findings of numerous intracellular and extracellular bacilli in the areas of the brain stem and cerebellar white matter [6,14,15].

## Conclusion

This case illustrates the importance of neuromonitoring in patients with severe brain damage due to a severe CNS infection. Despite prompt antibiotic and corticosteroid administration, listerial CNS infections can be lethal especially if focal neurologic signs are present.

## Abbreviations

CNS: central nervous system; CSF: cerebrospinal fluid; CT: computed tomography; ICU: intensive care unit; TCD: transcranial Doppler sonography.

## Consent

Written informed consent was obtained from the next-of-kin of the patient for publication of this case report and any accompanying images. A copy of the written consent is available for review by the Editor-in-Chief of this journal.

## Competing interests

The authors declare that they have no competing interests.

## Authors' contributions

DK performed the non-invasive neuromonitoring procedures in the ICU and drafted the manuscript. GS participated in the medical interventions and provided expert advice for the listerial infection. VG participated in all radiologic investigations and drafted the manuscript. AK participated in all medical interventions and drafted the definite version of this manuscript. All authors read and approved the final manuscript.
